# Polyesters Incorporating Gallic Acid as Oxygen Scavenger in Biodegradable Packaging

**DOI:** 10.3390/polym14235296

**Published:** 2022-12-04

**Authors:** Uruchaya Sonchaeng, Juthathip Promsorn, Nattinee Bumbudsanpharoke, Vanee Chonhenchob, Shyam S. Sablani, Nathdanai Harnkarnsujarit

**Affiliations:** 1 Department of Packaging and Materials Technology, Faculty of Agro-Industry, Kasetsart University, 50 Ngam Wong Wan Rd., Latyao, Chatuchak, Bangkok 10900, Thailand; 2 Center for Advanced Studies for Agriculture and Food, Kasetsart University, 50 Ngam Wong Wan Rd., Latyao, Chatuchak, Bangkok 10900, Thailand; 3 Biological Systems Engineering Department, Washington State University, Pullman, WA 99164-6120, USA

**Keywords:** active packaging, advanced material, food packaging, functional polymer, oxygen absorber

## Abstract

Biodegradable polyesters polybutylene succinate (PBS) and polybutylene adipate-co-terephthalate (PBAT) were blended with gallic acid (GA) via cast extrusion to produce oxygen scavenging polymers. The effects of polyesters and GA contents (5 to 15%) on polymer/package properties were investigated. Increasing GA formed non-homogeneous microstructures and surface roughness due to immiscibility. GA had favorable interaction with PBAT than PBS, giving more homogeneous microstructures, reduced mechanical relaxation temperature, and modified X-ray diffraction and crystalline morphology of PBAT polymers. Non-homogenous dispersion of GA reduced mechanical properties and increased water vapor and oxygen permeability by two and seven folds, respectively. Increasing amounts of GA and higher humidity enhanced oxygen absorption capacity, which also depended on the dispersion characteristics of GA in the matrices. PBAT gave higher oxygen absorption than PBS due to better dispersion and higher reactive surface area. GA blended with PBAT and PBS increased oxygen scavenging activity as sustainable active food packaging using functional biodegradable polymers.

## 1. Introduction

The food industry now promotes sustainable biodegradable packaging to mirror the environmental concepts of consumers. Global production of biodegradable polymers is increasing, particularly for flexible and rigid packaging applications. Disposal of non-biodegradable plastics causes pollution through waste accumulation. Several biodegradable polyesters, including polybutylene succinate (PBS), polybutylene adipate-co-terephthalate (PBAT), and poly(3-hydroxybutyrate-co-3-hydroxyvalerate) (PHBV), have been commercialized in the market [1,2,3,4,5,6,7]. However, biodegradable polymers have inferior barrier properties compared to synthetic conventional non-biodegradable plastics. Enhanced functionality of these materials compensates for poor barrier properties and can extend the shelf-life of packaged foods.

Oxygen scavenging active packaging has recently been developed to preserve food quality. However, iron-based inorganic oxygen scavengers have major drawbacks, including potential metal contamination [8], while powder applications are limited to beverages. Oxygen scavenging polymers have wider applications in active packaging, such as liners, which eliminate residual oxygen in package headspace. The current trend of natural products with green labels drives the development of packaging toward organic components as oxygen scavengers, including ascorbic acid, gallic acid (GA), and pyrogallol [8,9].

GA is a plant-based phenolic compound containing three hydroxyl (OH) groups bound to an aromatic benzene ring. It is highly soluble in water and has been used as a crosslinking agent and antioxidant to inhibit oxidative degradation in food [10} (Singh et al., 2021). GA efficiently absorbs oxygen when combined with alkaline substances, such as sodium carbonate, sodium hydroxide, or potassium chloride. GA contains four acidic protons, which form gallate anions depending on the pH of the medium. Autoxidation produces gallate radicals by the transfer of an electron or hydrogen atom, forming intermediates that can absorb oxygen [7,10,11,12,13]. The kinetics of oxygen scavenging have been demonstrated in GA powder and are enhanced by temperature and humidity [11]. Recent research development into active films containing GA as an oxygen scavenger includes low density polyethylene film [13], bio-based multilayer film [14], PHBV [7], and chitosan [10], demonstrating the potential of GA as a natural oxygen scavenger in the food and packaging industry.

Here, biodegradable oxygen scavenging polymers were developed using gallic acid and polyesters. Two common biodegradable polyesters with different characteristics, namely PBAT and PBS, were blended with GA via cast extrusion. PBAT has high flexibility characteristics, while PBS is more rigid polyesters due to higher degree of crystallinity. These bioplastic materials have been majorly used in the packaging industry. Our trial experiments showed that extrusion blending of GA with PLA, a major utilized biodegradable polyester, degraded PLA structures, giving liquid-like melt. In the present study, microstructures, morphology, thermal behavior, and packaging properties of different polyester structures and GA contents were investigated, and absorption capacity was determined at different relative humidity representing dried and intermediate moisture foods. It was hypothesized that adding GA into biodegradable polymers via extrusion enhanced oxygen absorbing capacity, while higher concentrations of GA increased oxygen absorption efficiency but caused non-homogeneous matrices, which subsequently reduced mechanical and barrier properties. Findings support commercial production via the extrusion process of organic-based oxygen scavenging biodegradable polymers for use in the food and packaging industry. Oxygen absorbing polymers can be used as internal bottle liners and inserts in high barrier packaging, such as glass and metal cans, to remove residual oxygen from the headspace and minimize oxidative reactions in packaged foods.

## 2. Materials and Methods

### 2.1. Materials

Gallic acid (GA) was purchased from Xi’an Ceres Biotech Co., Ltd. China. Potassium carbonate as reagent grade was sourced from KemAus™, Elago Enterprises Pty Ltd., Cherrybrook, N.S.W., Australia. Biodegradable polyester as polybutylene adipate-co-terephthalate (Ecoflex^®^F Blend C1200, BASF, Ludwigshafen, Germany) was purchased from Polymats Co., Ltd., Bangkok, Thailand, and PBS (BioPBS™ FZ91PM) was bought from PTT MCC Biochem Company Limited, Bangkok, Thailand.

### 2.2. Film Preparation

#### 2.2.1. Master Batch of Gallic Acid with PBAT or PBS

Biodegradable polyesters (PBAT and PBS) blended with GA were produced as a master batch via twin-screw extrusion. PBAT and PBS were dried in a hot air oven at 50 °C for 24 h to remove residual moisture. The plastic pellets were mixed with 50% (*w*/*w*) gallic acid and potassium carbonate at a 1:1 ratio using a dough mixer (HM-275; Otto King Glass Co., Ltd., Bangkok, Thailand) for 10 min (corresponding with 1:1:2 ratios of GA: potassium carbonate: PBAT or PBS). The mixtures were subjected to twin-screw extrusion (L/D ratio 40, screw diameter 35 mm, Model TSJ-35A, Ningbo Thaison Mold Plastic Co., Ltd., Zhejiang, China) using a screw speed of 185 rpm at 140 to 160 °C to achieve master batches of PBAT and PBS pellets containing 50% (*w*/*w*) GA and potassium carbonate blends.

#### 2.2.2. Cast Sheet Extrusion

PBAT or PBS films containing GA were produced using cast sheet extrusion (CF-W400, Chareon TUT, Samut Prakan, Thailand). Neat PBAT or PBS pellets were mixed with a master batch containing GA to achieve final contents of GA at 0, 5, 10, and 15% (*w*/*w*). The mixtures were physically blended using a dough mixer (HM-275; Otto King Glass Co., Ltd., China) for 10 min. The bioplastic mixtures were subjected to cast sheet extrusion at a temperature profile from 100 to 155 °C at 55 rpm screw speed. Incorporating different GA contents and polymer types (PBAT and PBS) slightly affected the processing efficiency. The temperature and screw speed of the extruder had been slightly adjusted to achieve continuous formation of plastic sheets. The screw configuration was the same for all formulations. Films were kept in vacuum-sealed aluminum bags.

### 2.3. Film Characterization

#### 2.3.1. Microstructure

The films were freeze-cracked by immersion in liquid nitrogen. Small pieces of film samples were placed on a stub using two-sided carbon tape and coated with gold using a sputter coater (SPI-Module, Structure Probe, Inc., West Chester, PA, USA). Surface and cross-section microstructures of the film samples were observed using a scanning electron microscope (JSM-6610LV, Jeol Ltd., Peabody, MA, USA) at 10 kV and magnification 200× (modified from Phothisarattana & Harnkarnsujarit, 2022 [15]).

#### 2.3.2. Surface Topography

Surface morphology of the films was investigated by atomic force microscopy (AFM) using an MFP-3D-Bio (AFM) (Asylum Research, USA). Topographic images represented 20 × 20 μm^2^ of film area. Tapping mode was performed to produce topographic images of 400 μm^2^ film area. The cantilever length was 225 μm with resonance frequency 190 kHz and nominal tip radius curvature of 8 nm at a scan rate of 0.8 Hz. Surface roughness was calculated from the root mean square average (Rq) of height deviations, taken as five samples from the mean data plane using Igor Pro version 6.37 software.

#### 2.3.3. X-ray Diffraction (XRD)

X-ray diffraction (XRD) patterns were determined using an X-ray diffractometer (D8, Bruker AXS, Germany) with range at 2θ from 4 ° to 40°, 40 kV voltage, 40 mA current at a rate of 0.8°/s, and a 0.02° step size [16].

#### 2.3.4. Dynamic Mechanical Thermal Analysis (DMTA)

Film samples were cut into 1 cm × 2 cm rectangular shapes. Mechanical relaxation was measured by a dynamic mechanical thermal analyzer (DMTA, Mettler Toledo-DMA1, Switzerland) and analyzed in tension mode between −100 to 100 °C. Frequencies used were 0.5, 1.0, 5.0, 10.0 and 20.0 Hz with heating scanning rate of 2 °C/min. Tan delta (tan δ) was reported for each sample [16].

#### 2.3.5. Mechanical Properties

The samples were cut into 2.5 cm ×10 cm strips in machine direction (MD) and cross direction (CD) and determined for five measurements of thickness using a digital micrometer. All samples were kept at 50%RH for 48 h before measurement. Mechanical properties (tensile strength, elongation at break, and Young’s modulus) were determined following ASTM D882-10 (ASTM, 2000) using an Instron universal testing machine. Distance between the handle was 5 cm with speed 500 mm/min. Tensile strength (TS), elongation at break (EB), and Young’s modulus (YM) values were averaged from ten measurements.

#### 2.3.6. Surface Hydrophobicity

Contact angle of the film samples was measured using an OCA 15EC contact angle analyzer (DataPhysics Instruments GmbH, Filderstadt, Germany) with SCA 20 software for data acquisition. Film samples were cut into a rectangular shape (1 cm × 10 cm) and placed on a stage at 50%RH for 48 h. Three microliters of distilled water were dropped on the films using a micro syringe connected to the computer system. The image was captured immediately. Contact angle data were averaged from ten samples (modified from Wongphan et al., 2022 [16]).

#### 2.3.7. Barrier Properties

##### Water Vapor Permeability

The three film samples were cut into 7 cm diameter circles and placed on metal cups containing dried silica gel. An O-ring was placed on the film edge and sealed using hot paraffin wax. The samples were kept at 25 °C and 50%RH and weighed every day until constant weight. Water vapor transmission rate (WVTR) was determined using a standard cup method (ASTM E96-80). Water vapor permeability (WVP) was derived from the slope of the linear weight increase and calculated as:

WVP=(WVTR × thickness) /Δp

where Δp is the water vapor partial pressure difference between both sides of the film calculated by saturated vapor pressure at 25 °C (S = 0.0313 atm) × difference in relative humidity (0.5).

##### Oxygen Permeability

Film samples were cut into 14 cm diameter circles and kept in a humidity chamber at 50%RH for 48 h before testing. Oxygen transmission rate (OTR) was determined as ASTM D3985-051 (ASTM, 2010) using an 8500 Model oxygen permeability analyzer (Illinois Instruments, Inc., Johnsburg IL, USA). Data were averaged from four samples and calculated for oxygen permeability (OP) as:

OP = (OTR × *L*)/*Δp*

where *L* is the mean sample thickness and *Δp* is the oxygen partial pressure difference between the two sides of the sample.

### 2.4. Oxygen Absorption Capacity

The film samples were cut into 5 cm × 5 cm squares and kept in a clear 480 mL glass bottle with ambient air (20.6%, O_2_) using saturated salt solution, namely magnesium nitrate (Mg(NO_3_)_2_) and sodium chloride (NaCl), to generate different relative humidity at 50 and 75%RH, respectively, before storage at 25 °C. The oxygen content was measured using a gas headspace analyzer (Dansensor^®^ CheckPoint3, Dansensor, Ringsted, Denmark) with gas components collected using a needle connecting with the headspace of the glass bottle through the septum. The data were averaged from duplicate samples.

### 2.5. Statistical Analysis

Statistical analysis was performed by one-way analysis of variance (ANOVA) using SPSS version 26 (SPSS Inc., Chicago, Illinois, USA). Significant differences among samples were determined using Duncan’s multiple range tests at a confidence interval of 95% (*p* ≤ 0.05).

## 3. Results and Discussion

**Figure 1:**
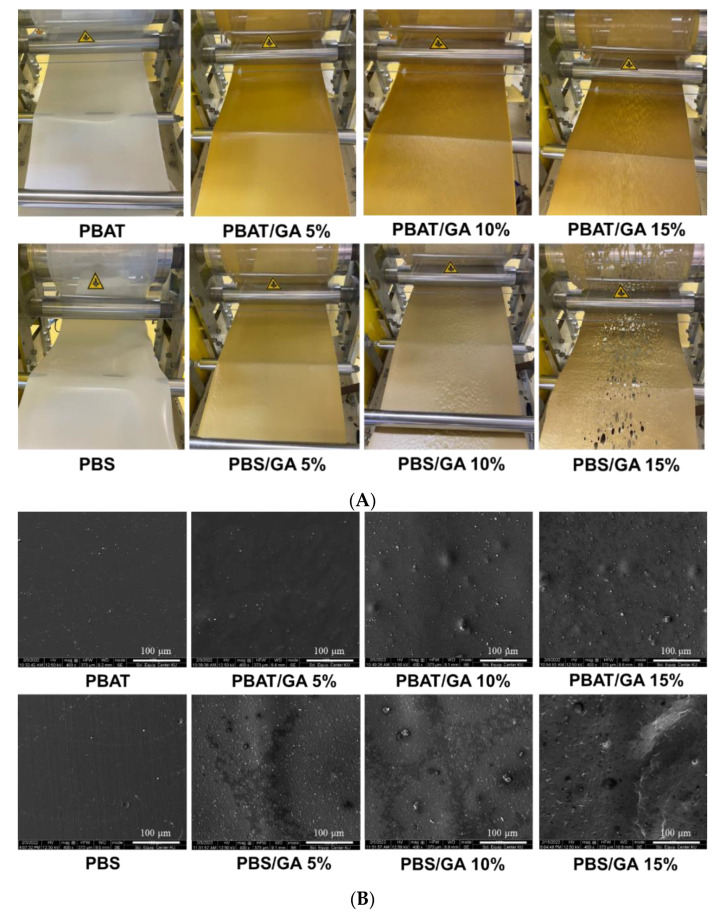

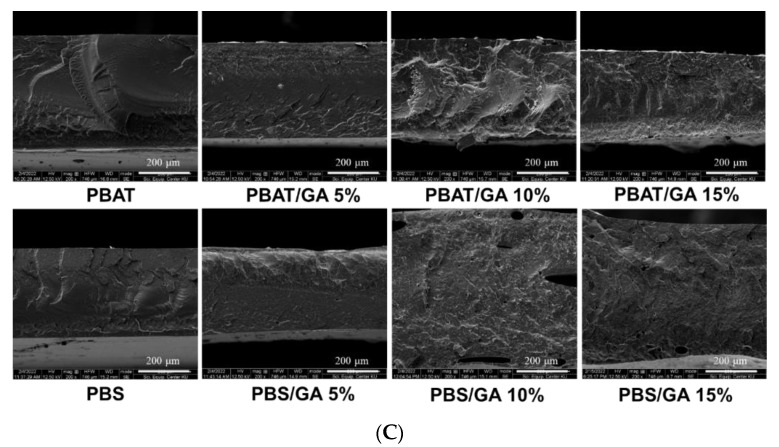
(**A**) Appearance of film during extrusion casting and SEM microstructures. (**B**) Surface and (**C**) cross-sections of PBAT and PBS films containing gallic acid (GA) at 0, 5, 10, and 15%.

PBAT and PBS films showed white opaque characteristics, while adding GA at 5% caused homogeneous yellowish color (Figure 1A). Higher GA content resulted in a non-homogeneous appearance and darker color. PBS containing GA 15% could not form continuous polymer film networks because high amounts of GA caused phase separation, which hindered network formation. PBS networks were more rigid than PBAT due to rapid crystallization during cooling from the molten state [4,17]. This reduced flexibility and negatively impacted structural formation during the casting process of PBS/GA15. Adding more GA at and above 15% in PBS caused non-homogeneous film structures with breakage. The film cannot continuously form during cast extrusion. Conversely, PBAT showed high ability to dispersed GA, probably due to higher amorphous polymer contents to interact with GA.

### 3.1. Microstructures

Surface microstructures of the PBAT and PBS films are shown in Figure 1B. Neat polymers had smooth structures, while increasing GA content resulted in non-homogeneity. PBAT films containing GA had a smoother surface. PBS typically had a rougher surface with fine voids (space) due to rapid crystallization at the surface forming polymer crystallites [17,18]. Cross-section microstructures also showed smooth structures in neat PBAT and PBS (layer structures attributed to specimen preparation by freeze-cracking) as shown in Figure 1C. Adding more GA, particularly 10 and 15%, gave non-homogeneous structures, indicating phase separation between GA and the polymers. GA did not form a network structure and, instead, presented as a dispersed phase in continuous polymer matrices. Miscible polymer blends showed smooth morphology [19]. At low concentrations (5% GA), miscibility of GA in the polymers gave a smooth structure desirable for consumers. PBS containing GA 10 and 15% showed void spaces in cross-section microstructures. Results showed that PBAT was more compatible with GA than PBS and formed a homogeneous network structure.

### 3.2. Surface Topography

**Figure 2:**
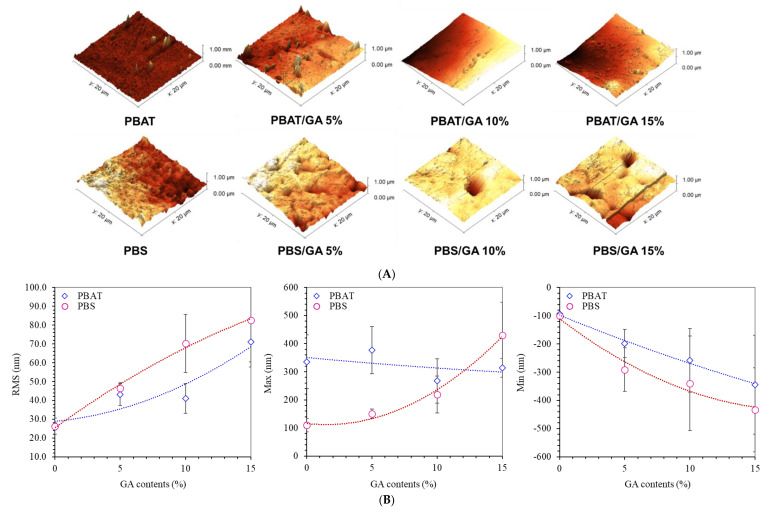
AFM analysis: (**A**) Topographic images and (**B**) surface roughness as root mean square (RMS), maximum and minimum surface height of PBAT and PBS films containing gallic acid (GA) at 0, 5, 10, and 15%.

AFM reflects surface roughness which affects surface properties. Topographic images show smoother surfaces in PBAT than PBS (Figure 2A). Rapid crystallization of PBS caused heterogeneous structures of embedding crystallites [4]. PBS films containing GA had void spaces with size around 1–5 μm. Root mean square (RMS) surface roughness indicated variation of surface peak height (Figure 2B). RMS values increased with higher GA content for both PBAT and PBS films. PBAT had lower RMS values due to the smoother surface. Increasing GA caused high numbers of immiscible particles which increased variations in surface height, giving deeper valleys and higher peaks, particularly in PBS matrices (Figure 2B). Conversely, maximum peak height for the PBAT phase was insignificantly affected by GA content. GA clumps were formed and embedded beneath the surface of flexible PBAT networks. The flexible structure of PBAT enhanced dispersion of GA particles more than rigid networks in PBS.

### 3.3. X-ray Diffraction

**Figure 3:**
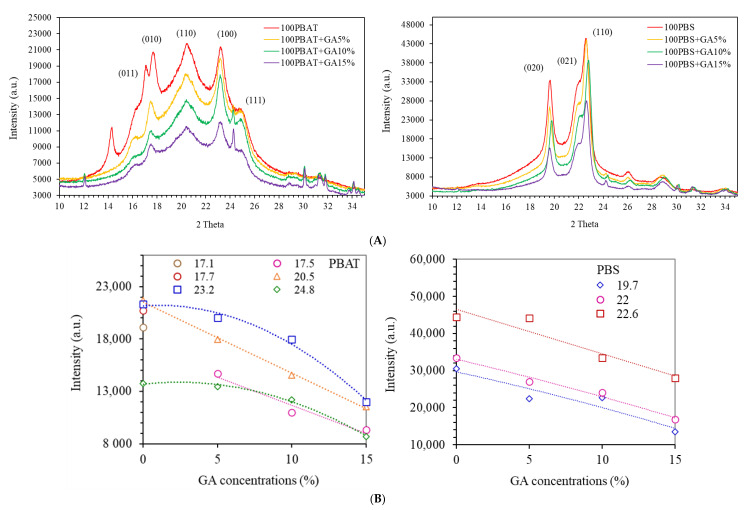
X-ray diffraction (XRD) analysis: (**A**) diffractograms and (**B**) diffraction intensity at different 2θ values of PBAT and PBS films containing gallic acid (GA) at 0, 5, 10, and 15%.

Crystallinity influenced both the physical and barrier properties of the films [20]. X-ray diffraction patterns of PBAT and PBS films are shown in Figure 3A. PBS had much higher XRD intensity than PBAT systems, confirming the higher ability to crystalize after solidification into amorphous matrices. Low glass transition temperature (*T*_g_ ~ −30 to −35 °C) with a linear chain readily enhanced nucleation and growth of crystallites. Crystallization of PBS molecules from melt first forms α-crystals, which then further transform into β-crystals due to applied stress, with monoclinic unit cells in both crystal structures [21]. Cell dimensions between α-crystals (a = 0.523 nm, b = 0.912 nm, c = 1.090 nm) and β-crystals (a = 0.584 nm, b = 0.832 nm, c = 1.186 nm) are different [21,22]. PBS and PBS with GA showed diffraction planes corresponding with (020), (021), and (110) lattices. The intensity of XRD peaks reduced linearly with increasing GA contents due to reducing ratios of crystalline PBS fraction (Figure 3B). Slope of reduction was also identical in all lattice forms. Results suggested that GA had insignificant effects on crystalline lattice structures of PBS polymers.

PBAT showed typical diffraction at planes (011), (010), (110), (100), and (111) corresponding with different lattice diffraction angles (Figure 3A). Adding GA gave rise to the peak at 2θ = 24.3° due to crystals of carbonate lattice in the film formulations [23]. GA also modified the diffraction peaks at 2θ = 17.1° and 17.7° in PBAT structures. Diffraction peaks at these two angles were eliminated, while a new peak at an intermediate angle of 2θ = 17.5° was predominant. Shifting of the diffraction angles probably resulted from strong interaction between the carbonyl group of PBAT with the hydroxyl group of GA that modified intercalation in PBAT chain molecules [1]. Results reflected the interaction between GA and PBAT structures, which altered lattice structures involving (011) and (010) planes. Intensity of PBAT diffractions at different 2θ are shown in Figure 3B. Diffraction angles at 2θ = 17.5° and 20.5° linearly decreased with increasing GA, suggesting that further increasing GA had no effect on crystalline structures. However, non-linear correlations between XRD intensity and GA concentrations were found for diffraction peaks at 2θ = 23.2° and 24.8°, suggesting modified crystal lattice in planes (100) and (111). The findings suggested interaction between PBAT and GA, which altered the crystalline polymorphs of PBAT. It should be noted that the infrared spectra of biodegradable PBAT and PBS are further performed to clarify chemical interaction between gallic acid and these polyesters. The XRD diffractograms also reflected different roles of GA on morphologies of these polyesters, consisting of aliphatic structures (PBS) and aromatic ring (PBAT) connected butylene.

### 3.4. DMTA

**Figure 4:**
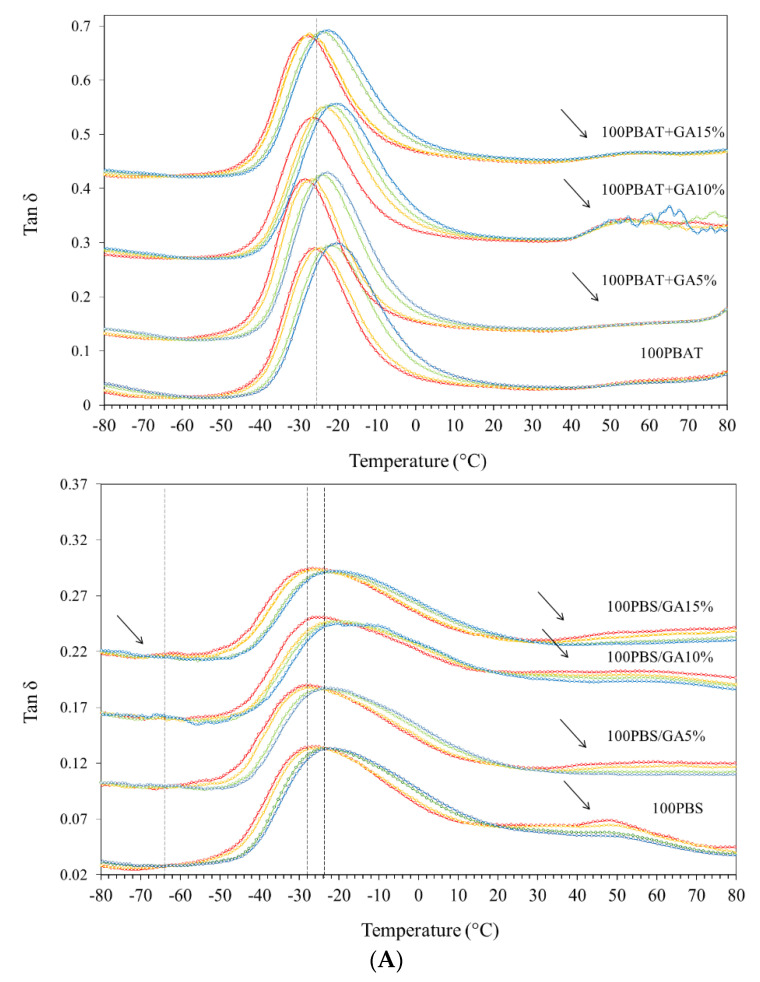

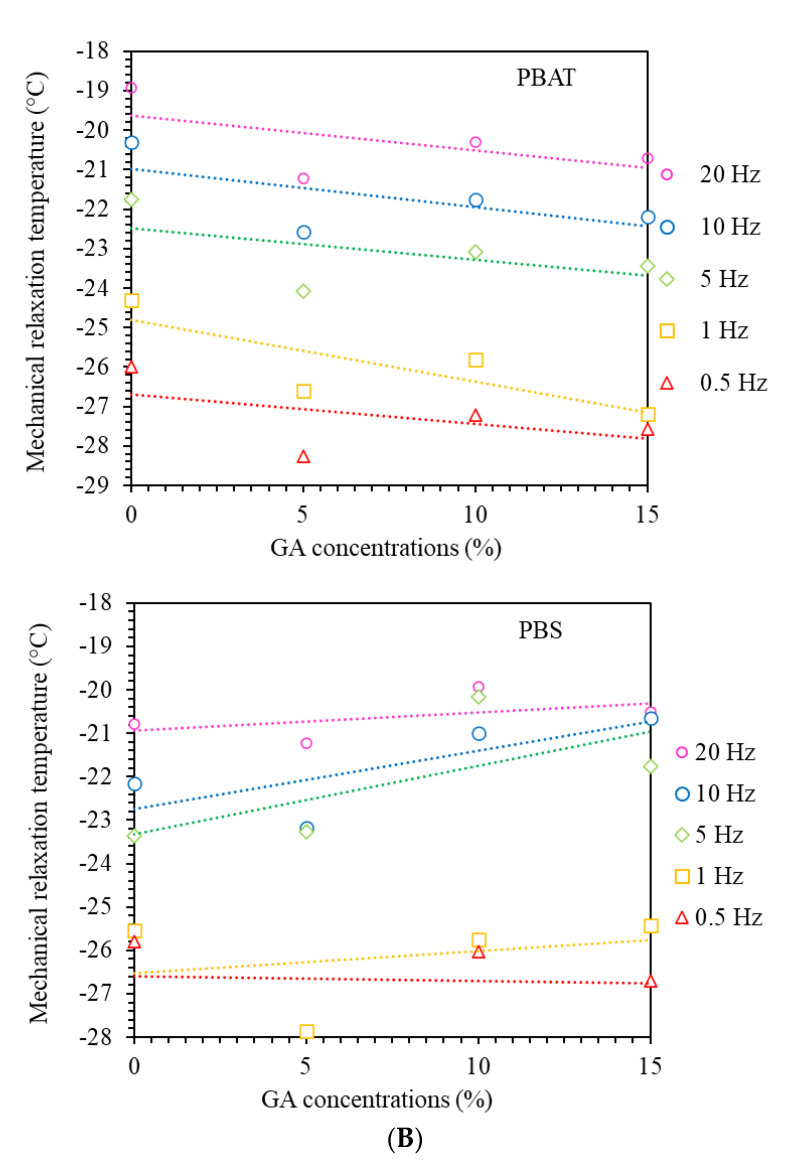
Dynamic mechanical thermal relaxation analysis: (**A**) Tan δ values and (**B**) mechanical relaxation temperature of PBAT and PBS films containing gallic acid (GA) at 0, 5, 10, and 15% and diverse frequencies as 0.5, 1, 5, 10, and 20 Hz.

Tan δ is the ratio between loss and storage modulus and measures the dissipation energy of a material. Mechanical relaxation as tan δ peaks at different frequencies or “α-relaxation” involving *T_g_* of amorphous polymers are shown in Figure 4A. Height of relaxation peaks decreased with decreasing polymer contents (replacement by GA) due to decreasing segmental mobility [24]. PBAT had stronger magnitude (peak height) of α-relaxation than PBS (by approximately two folds), possibly due to more segmental mobility of polymer components containing aromatic benzene in the adipate-co-terephthalate unit. The presence of aromatic structures enhanced energy dissipation leading to a higher tan δ peak [25]. Conversely, linear structures of PBS molecules facilitated closer packing of the chains. Strong matrix interaction and immobilization of polymer molecules gave lower tan δ peak height [26]. The peak of tan δ in PBAT was located at a higher temperature than PBS (~2 °C), while the initial rising temperatures were −58 °C and −64 °C for PBAT and PBS, respectively. An initial increase in tan δ suggested higher onset temperature with increasing molecular mobility and a higher tendency for translational mobility to form crystals in the PBS phase. PBS has a linear structure, while PBAT contains a bulky aromatic group acting as a steric hindrance for crystallization.

Tan δ of a glassy material is low due to restricted mobility of the chain segments and the applied thermal energy was insufficient to enhance segmental motion of the polymers, while energy dissipation or damping was highest during glass transition because of increased chain mobility [26,27]. Peaks of tan δ were derived for α-relaxation temperature (*T_α_*) as shown in Figure 4B. Adding GA had insignificant effects on *T_α_* of PBS, while PBAT showed decreased *T_α_* (up to ~2 °C) with GA. Modification of *T_α_* was attributed to the decreasing T_g_ of PBAT due to interaction with GA, which plasticized and increased the free volume of the polymers. The presence of aromatic rings in both PBAT and GA transferred local electrons, forming chemical bonding in the amorphous phase.

### 3.5. Mechanical Properties

High strength and proper elongation are required for flexible packaging materials. Tensile properties of PBAT and PBS films (tensile strength, TS; elongation at break, EB; and Young’s modulus, YM) are shown in Table 1. PBS with 15% GA could not form continuous polymer film networks in extrusion casting and was eliminated from further measurements. PBS was more rigid and less flexible, giving higher TS and YM, with lower EB than PBAT. Crystallization of PBS gave low network deformability with EB values of 35%. Adding GA sharply decreased TS and EB in both polymers due to non-homogeneity of the film matrices [27]. Transport of stress through the matrices directly influences the strength of polymeric networks. More homogeneous and connecting structures give higher area for better stress transfer [28]. Conversely, the presence of foreign rigid particles form voids due to poor adhesion at the interface (GA fillers and polymers). This hinders stress distribution, with reduction in mechanical properties [29,30].

The machine direction (MD) gave higher TS than cross-direction (CD) in neat films (both PBAT and PBS) (Table 1) due to the orientation of polymer molecules that enhanced network strength. Conversely, EB values were identical for MD and CD in neat films, suggesting that orientation had an insignificant effect on film extensibility. Stretching induced molecular orientations of polymer chains, which improved matrix strength; however, elongation also requires the ability to deform under applied external stress. Facca et al. (2007) [28] indicated that reduction in the stress transfer area corresponded with increased brittleness of polymers. Differences between MD and CD were more pronounced at higher film GA contents. The presence of foreign solid particles in film matrices impeded polymer bonding. MD retained better mechanical strength, suggesting that stretching in extrusion facilitated the dispersion of GA particles. Cui et al. (2021) [31] also indicated that film stretching enhanced the dispersion of filler particles in polymeric films.

### 3.6. Surface Hydrophobicity and Barrier Properties

Surface hydrophobicity controls the wetting properties of liquid on the film surface and was determined by contact angle (CA) (Table 1). Higher CA is desirable to reduce water sensitivity of the film surface. Neat PBS had a higher CA than PBAT, corresponding with higher surface roughness which gave lower wettability of the hydrophobic surface [32]. However, surface hydrophobicity decreased with increasing GA contents in both PBAT and PBS due to increasing hydrophilicity by GA, which contains hydroxyl groups and readily absorbs water [10]. PBS showed a sharp decrease in CA when adding 5% GA, while PBAT showed linear decrease in CA with increasing GA contents. Reduced interaction between GA and PBS possibly enhanced phase separation and aggregation of GA clumps, which readily exposed and bonded with the water droplets.

High barrier against diffusion of water vapor is a desirable characteristic to preserve the quality of packaged foods. Water vapor permeability (WVP) of PBAT was higher than PBS by approximately 3 folds (Table 1). Lower crystallinity of PBAT and higher free volume of the polymer allowed higher diffusion of water vapor. WVP increased with increasing GA in PBAT films possibly due to the hydrophilicity of the matrices. PBS with 5% GA had identical WVP to neat films. High degree of crystallinity in PBS films effectively limited diffusion of water vapor at low GA of 5%. Increasing GA to 10% enhanced hydrophilicity and significantly increased WVP. Conversely, Pacheco et al. (2019) [33] showed that adding GA to chitosan films reduced WVP by approximately 60% due to hydrogen bonding between polyphenols and biopolymers, as well as hydrophobic interactions which limited the interaction of free OH groups with water. Similarly, Singh et al. (2021) [10] found reduction in WVP in chitosan films with addition of GA produced by solvent casting techniques. PBAT and PBS formed fewer hydrogen bonds with GA (compared with chitosan), giving larger numbers of free hydroxyl groups in phenolic GA structures. Previous investigations produced films via solvent casting techniques. GA fully dissolved in the solvent and homogeneously dispersed in amorphous chitosan film. In this study, GA formed clumps due to solventless extrusion casting. The non-homogeneity of the matrices and poor adhesion at the GA-polymer interface caused void spaces, allowing higher diffusion. These diverse results suggested that interaction between polymer matrices and GA has a major effect on permeability.

Oxygen permeation influences the quality of packaged foods, particularly the stability of oxygen-sensitive products e.g., lipids, vitamins, and nutrients. Oxygen permeability (OP) of the films is shown in Table 1. Neat PBS had lower OP than PBAT due to higher crystallinity and, hence, more tortuous paths for gas diffusion [4]. However, adding GA sharply increased OP values in PBS films. AFM results also indicated increasing maximum peak height and deeper valleys in PBS with higher GA contents. Incompatibility between GA and PBS enhanced non-homogeneity of the matrices, forming void spaces between the immiscible interface. Two oxygen atoms share the same number of electrons and form diatomic oxygen molecules, which are non-polar. The transport of oxygen is enhanced by the hydrophobicity of the matrices [34]. High affinity between oxygen and PBS efficiently enhanced diffusion of gas through hydrophobic matrices in the amorphous PBS phase. Conversely, PBAT had higher dispersion of hydrophilic GA molecules throughout the polymer network. Hydrophilic layers of dispersed GA delayed the transport of non-polar oxygen molecules due to their reduced affinity at the interface [26,35]. Accordingly, adding GA at 5% gave identical OP to neat PBAT. Further increasing GA content significantly increased OP due to non-homogeneity, which formed higher numbers of voids at the interface, allowing higher rates of gas diffusion. Higher permeation of the films can be useful for packaging of fresh produce in which gas permeation and water vapor transfer are required to allow aerobic respiration and avoid accumulation of water condensate. Results indicated the complex roles of morphology on the permeability of volatiles, including crystallinity, hydrophilic-hydrophobic structures, and void spaces.

### 3.7. Oxygen Absorption Capacity

**Figure 5:**
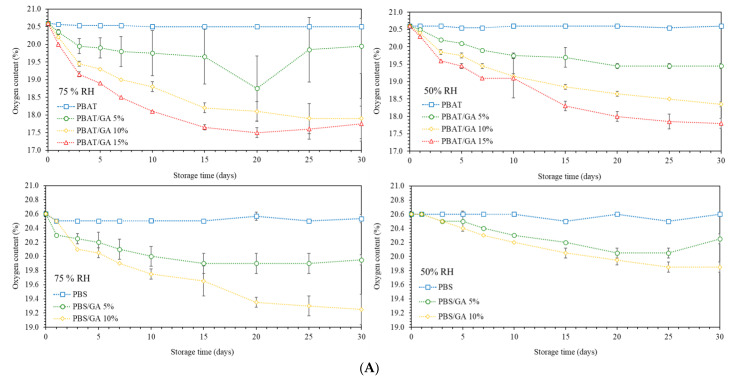

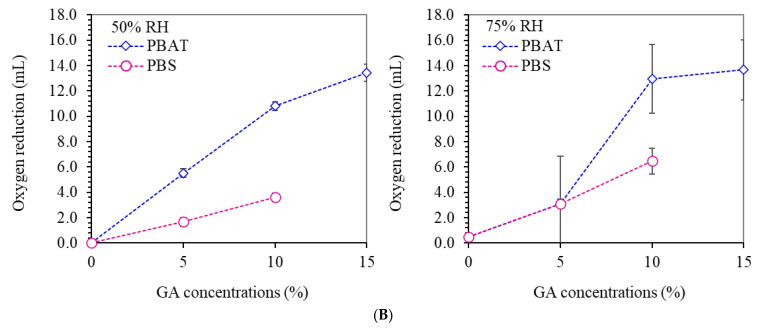
Oxygen scavenging activity of PBAT and PBS films containing gallic acid (GA) at 0, 5, 10, and 15% (**A**) Oxygen levels as a function of time and (**B**) volume of oxygen reduction under storage at 50% and 75% RH at 25 °C.

Oxygen accelerates quality deterioration of several foods, and removal of oxygen is necessary for oxygen-sensitive products. Removal of oxygen from the packaged headspace possibly inhibits aerobic microorganisms, replacing addition of food preservative. Films were determined for oxygen absorption capacity at different relative humidity (RH 50% and 75%) at 25 °C (Figure 5A). Adding GA to the polymers effectively reduced oxygen contents in the package headspace and higher levels of GA loading improved oxygen absorption capacity. GA substances can react with oxygen molecules in the presence of potassium carbonate [7,14] and, therefore, the films potentially reduced oxygen levels in the packaging. PBAT/GA films gave lower oxygen levels than PBS/GA at identical GA concentration. Higher dispersion of GA in PBAT gave smaller particle size and a larger surface area to react with oxygen, providing higher oxygen scavenging capacity than PBS. Conversely, larger GA clumps in the PBS matrix reduced the surface area for oxygen exposure. Singh et al. (2021) [10] indicated maximum oxygen scavenging capacity of chitosan films containing 1, 5, 10, and 20% GA as 2, 4, 11, and 20 mL O_2_/g, respectively, at 23 °C, while Di Giuseppe et al. (2022) [7] showed oxygen absorption of GA incorporated poly(3-hydroxybutyrate-co-3hydroxyvalerate) active films as 120 mg O_2_/ g GA at room temperature.

Increasing the relative humidity boosted oxygen reduction due to enhanced reaction rates. Figure 5B shows the amounts of oxygen reduction. PBAT/GA 15% gave maximum oxygen reduction by 13.4 mL and 13.7 mL at 50%RH and 75%RH, respectively. At 50% RH, PBS gave lower oxygen absorption capacity than PBAT by 3.0–3.3 folds. Increasing the humidity to 75%RH reduced the difference between PBAT and PBS film capacity to only two folds. Similarly, Pant et al. (2017) [14] indicated that higher RH significantly improved oxygen absorption rates of GA-based packaging. Increasing humidity induced water interaction with GA, which enhanced swelling of the clumps that readily reacted with oxygen molecules. Water also activated and increased molecular mobility of the amorphous hydrophilic GA and carbonate solids, which catalyzed oxygen reduction [36]. Results indicated that oxygen scavenging capacity depended on the amount and dispersion characteristics of GA in the matrices and was enhanced by humidity. These findings clearly proved that extrusion compounding of gallic acid with biodegradable PBAT/PBS polyesters as masterbatch efficiently provides oxygen scavenging capability to bioplastic materials. The proposed protocol is convenience for plastic manufacturing, which can be further useful for scaling-up processing and commercial production of active functional polymers.

## 4. Conclusions

Biodegradable polyester films (PBAT and PBS) containing gallic acid efficiently acted as oxygen scavenging polymers to remove oxygen in packaging. At a low concentration of 5%, GA homogeneously dispersed in polyester, giving a slight yellowish film color. Increasing amounts of gallic acid formed clumps due to immiscibility with the polymers and caused non-homogeneous microstructures, particularly in PBS. PBAT showed smoother microstructures and surface topography, while PBS mixed with gallic acid showed reduced homogeneity with the occurrence of void spaces and higher surface roughness. Gallic acid modified X-ray diffraction angles of PBAT, suggesting interaction with polymers forming different crystalline morphology. The amount of gallic acid in the films influenced oxygen absorption capacity, which depended on dispersion characteristics. Enhanced dispersion of GA in PBAT gave higher oxygen absorption capacity than PBS. Incorporating GA into biodegradable PBAT and PBS films via cast-extrusion effectively produced functional active packaging.

## Figures and Tables

**Table 1:** Mechanical properties as tensile strength (TS), elongation at break (EB), Young’s modulus (YM), contact angle (CA), water vapor permeability (WVP), and oxygen permeability (OP) of PBAT and PBS films containing gallic acid (GA) at 0, 5, 10, and 15%. Different letters indicate significant differences within the same column (*p* ≤ 0.05).

	TS (MPa)		EB (%)		YM (Mpa)		CA	WVP	OP
	MD	CD	MD	CD	MD	CD	(°)	(g.mm/m^2^.Day.atm)	(cm^3^.mm/m^2^.Day.atm)
PBAT	26.9 ± 0.4 ^e^	21.9 ± 0.6 ^e^	684.3 ± 100.0 ^b^	1108.7 ± 0.4 ^d^	89.3 ± 1.8 ^a^	99.5 ± 5.8 ^b^	79.4 ± 2.5 ^f^	3.164 ± 0.011 ^c^	50.7 ± 1.6 ^b^
PBAT/GA5%	22.6 ± 0.9 ^d^	20.3 ± 0.4 ^d^	1103.5 ± 6.1 ^d^	1096.3 ± 16.0 ^d^	83.9 ± 1.9 ^a^	78.8 ± 3.1 ^a^	73.9 ± 0.6 ^e^	3.201 ± 0.021 ^c^	52.8 ± 4.1 ^b^
PBAT/GA10%	15.9 ± 1.2 ^b^	8.0 ± 0.3 ^b^	1064.6 ± 33.1 ^d^	497.4 ± 53.3 ^c^	79.9 ± 13.1 ^a^	75 ± 1.9 ^a^	71.6 ± 1.1 ^d^	4.215 ± 0.258 ^d^	90.4 ± 16.9 ^c^
PBAT/GA15%	10.7 ± 1.3 ^a^	5.7 ± 0.7 ^a^	931.5 ± 62.6 ^c^	271.1 ± 98.0 ^b^	74.6 ± 4.3 ^a^	71.4 ± 4.4 ^a^	67.8 ± 0.8 ^c^	4.589 ± 0.445 ^d^	195.4 ± 6.3 ^e^
PBS	35.4 ± 0.9 ^f^	33.3 ± 0.9 ^f^	34.9 ± 4.7 ^a^	33.1 ± 7.1 ^a^	531.4 ± 9.8 ^d^	536.6 ± 15.8 ^e^	83.5 ± 2.9 ^g^	1.221 ± 0.052 ^a^	30.1 ± 1.3 ^a^
PBS/GA5%	25.8 + 0.8 ^e^	20.0 ± 0.6 ^d^	10.8 ± 0.6 ^a^	7.5 ± 0.5 ^a^	453.3 ± 12.0 ^c^	461.0 ± 6.9 ^d^	64.4 ± 1.1 ^b^	1.228 ± 0.142 ^a^	170.9 ± 18.1 ^d^
PBS/GA10%	18.8 ± 1.7 ^c^	10.1 ± 0.6 ^c^	8.8 ± 0.4 ^a^	5.3 ± 0.3 ^a^	405.5 ± 24.4 ^b^	309.1 ± 17.4 ^c^	60.6 ± 1.8 ^a^	2.186 ± 0.888 ^b^	208.2 ± 2.8 ^e^

## Data Availability

The data presented in this study are available on request from the corresponding author.
